# Institutional Notes and News

**Published:** 1910-11-05

**Authors:** 


### INSTITUTIONAL NOTES AND NEWS.

**Chesterfield and North Derbyshire Hospital.**

SINCE 1907 the authorities of the Chesterfield and North Derbyshire Hospital have been hard at work attempting to bring the institution up to date. The result of these efforts has been that the hospital accommodation has risen from eighty beds to one hundred and twenty, and any argument that this was extension rather than improvement is answered readily by the fact that during the last quarter the average of occupied beds has been 100.31. These improvements have cost £20,000, of which £13,000 has been raised, and have not been undertaken carelessly or without forethought, for so capable an authority on hospital construction and administration as Mr. W. G. Carnt, of Manchester Royal Infirmary, has been called in to advise and inspect the operations. His last visit took place a few days ago—the day before a meeting was held at which the Duke of Devonshire was the principal speaker, to appeal for money to clear off the debt. Mr. Carnt was prevented from attending the meeting, but he wrote a letter, which was read by Mr. Barnes, the Chairman of the Board of Management, from which we quote his own account of his visit: "I have visited the Institution during the progress of the work, and yesterday I had a further opportunity of inspecting the whole of the alterations, and I must heartily congratulate the committee and architect upon the result of their labours." This is especially noteworthy from the fact that Mr. Carnt, when first called in about five years ago, admitted that the prospect of reconstruction was not at first encouraging. The Duke of Devonshire, who again has given generously to the extension fund, not only made a capital speech at the meeting, but before that took place went over the wards and nurses' home, and was very pleased with what he saw. The extensions consist, briefly, of a new drainage system, enlargement of the main kitchen, an operation theatre, electric passenger lift, electric light installation, open-air balconies and outside stairs, extensive improvements in the administration block, a new nurses' home, "practically perfect" Mr. Carnt says, and, of course, the new wards and ward kitchens. In the face of the work done, and the authoritative praise for it, we cannot believe that there will be any difficulty in raising the necessary money to pay the remaining cost.

**Addenbrooke's Hospital, Cambridge.**

WE are glad to learn that the Cambridge local memorial to the late King is to take the form of a new out-patient department and of new wards for children at Addenbrooke's Hospital. More than once in these columns attention has been drawn to the need for extension at Addenbrooke's, and the public appreciation of this need at Cambridge is a healthy sign of the attachment of the University town to the hospital. It is, indeed, a startling proof of the public spirit of Cambridge in this matter that £4,000, in the words of the Mayor, Alderman W. P. Spalding, was promised before a penny had been asked for by him. The meeting which supported the memorial, the idea of which, we believe, the Mayor originated, was very influential and exceedingly enthusiastic, and its success has been increased by the shilling fund which the local newspaper at once opened. The sum needed to complete the two parts of the scheme is estimated at £15,000, of which £6,000 has been raised in the first week. It may be worth while to point out how badly a new children's ward is needed, since the only accommodation for children which the hospital has at the moment is a small ward which generally contains eight infants. The rest of the children have to be accommodated in the adult ward. As regards the out-patient department, Professor Howard Marsh emphasised the fact that it needs above everything else proper ventilation, good light, and plenty of space; these qualities, he added, were absent in Addenbrooke's hall. Perhaps we should add that the £166, which we reported in a recent issue to have been received last quarter from the Workers' Hospital Fund, was the result of money collected from about twenty-eight centres only. There are nearly seventy centres in all, and the movement has been so successful that it is probable that this number will have been doubled by next August.

**Hospital for Women, Liverpool.**

THE annual report of the Hospital for Women, Shaw Street, Liverpool, reveals a threatening deficiency of £920, or, we regret to add, nearly twice the deficiency that last year confessed to. In the face of the efforts that are now being made locally to clear off this debt, the hospital may hope for better times, and the quantity of work done during the past year has a more than usually topical interest. Over three thousand women have sought relief at the hospital, of whom 2,123 are out-patients. The hospital's most valuable work, however, is done probably indoors. We draw this distinction on the general ground that patients often apply at the out-patient department when delay has made matters worse than they might have been, and that the hospital has no means of pursuing into the homes, or grasping the conditions of work or unemployment which complicate each case. The paramount importance, now beginning to be recognised, of keeping the present and prospective mothers of the nation free from disease emphasises beyond doubt the importance of the work which the Women's hospital is undertaking. We hope that Liverpool will not be slow to appreciate its value.

**The New Norwich Infirmary.**

THE decision of the Norwich Board of Guardians in March 1908 to build a new infirmary has been fulfilled by the opening of the buildings last week in the presence of Mr. J. S. Davy, of the Local Government Board, the Lord Mayor, and the Dean of Norwich. The following is an excerpt of the architects' description (Messrs. Morgan and Buckingham): "The building is erected on the west of the existing old infirmary, and contains the administrative block and the women's pavilion, the old infirmary building being used at present as a men's pavilion. The whole design, when completed, with a new men's pavilion, will accommodate 215 patients, including 110 women and 105 men, and a resident medical officer. The administrative block contains, on the ground floor: Entrance hall, offices for resident medical officer and superintendent nurse, receiving-rooms for men and women, dispensary, anæsthetic, operating, and sterilising rooms. The second floor contains a suite of rooms for the resident medical officer, and store-rooms; and the third floor contains the maternity wards. The women's pavilion is connected to the administrative block by a corridor, the three floors having the same plan, which includes on each floor a large ward for thirty-two beds, a single-bed ward and a two-bed ward, a day-room, duty room for charge nurse, linen-store, bathroom and lavatories, and small stores. A large balcony is arranged on each floor."

**Aberdeen City Hospital.**

The enlargement of pavilions 1 and 4 at the City Hospital, Aberdeen, which increases the hospital's accommodation by thirty-four beds, has now been opened. The work has taken nearly two years to complete, and nurses and patients alike gain by the additional space allotted to them, which amounts to twelve extra bedrooms for the former and a pavilion for cases of skin-disease for the latter. Pavilion No. 4 of course has been built especially for phthisis cases, and is happy in a verandah screened with glass from the east. Lord Provost Wilson, who opened the pavilions, must have been very satisfied with the care expended on the work, and we hope the public will allow the increased accommodation to be used to the full by supplying the additional maintenance money which will be necessary if the patients are to have full benefit from the added number of beds.

**University College Hospital.**

We learn that over a thousand pounds has been received or promised as the result of the dinner which was held a few days ago to help the finances of University College Hospital. It will be remembered, as Mr. J. T. Bryan, the Mayor of St. Pancras, who presided, reminded the company, that the income must be raised by £6,000 if the hospital is to pay its way, and that early this year it was feared that 100 beds would be closed. This disaster was avoided, but it will be avoided permanently only if annual subscribers come forward and form an endowment for the splendid institution which Sir Blundell Maple built. It would seem that University College Hospital, on the design and bricks of which so much care and money were spent, should make an irresistible appeal to the public, for no thinking man can consent to see such a magnificent equipment paralysed by want of funds to keep it going. The demands made on the hospital are very great; 150,000 patients were treated there last year. Only one thing is needful—an annual income. This will come, however, if sufficient efforts are made to secure it.

**Leicester Maternity Hospital.**

At a public meeting in support of this hospital, held at the County Assembly Rooms, Mr. T. Cope, M.A., J.P. (Chairman of the County Council), presided, outlined the scheme of the hospital, and explained that its work covered the training of midwives as well as its maternity side. The hospital was opened in 1905 to accommodate five patients, and enlarged in 1907 to twenty-two beds. In 1906 thirty-six in-patients were received, which number quickly rose in 1909 to 255, while during 1910 they had received 240 patients. The number of midwives in the county before the passing of the Midwives Act was 370; there were now 110. Sir Francis H. Champneys, Bart. (Chairman of the Central Midwives Board), Mrs. Wallace Bruce (Association for Promoting Training and Supply of Midwives), Miss Robinson (Midwives' Institute), Dr. Thomas Wilson (Birmingham), and Dr. Killick Millard (Medical Officer of Health, Leicester) also addressed the meeting, which concluded with votes of thanks to the Chairman and the Mayor of Leicester for their attendance, on the motion of Mr. J. E. Faire, J.P. (Treasurer of the hospital), with Mr. J. M. Gimson (Chairman of the Executive Committee) as seconder. When we consider the increasing public recognition of the importance of healthy mothers for a healthy nation, the growing prestige of midwives and of maternity work is a sign to be thankful for. Leicester, we feel sure, appreciates this, and will not allow its maternity hospital to suffer from want of support.

